# Microbial Response to the MC-252 Oil and Corexit 9500 in the Gulf of Mexico

**DOI:** 10.3389/fmicb.2012.00357

**Published:** 2012-10-11

**Authors:** Romy Chakraborty, Sharon E. Borglin, Eric A. Dubinsky, Gary L. Andersen, Terry C. Hazen

**Affiliations:** ^1^Department of Ecology, Earth Science Division, Lawrence Berkeley National LaboratoryBerkeley, CA, USA; ^2^Department of Microbiology, The University of TennesseeKnoxville, TN, USA

**Keywords:** MC-252, oil, biodegradation, Corexit 9500, hydrocarbon, dispersant, Gulf of Mexico

## Abstract

The Deepwater Horizon spill released over 4.1 million barrels of crude oil into the Gulf of Mexico. In an effort to mitigate large oil slicks, the dispersant Corexit 9500 was sprayed onto surface slicks and injected directly at the wellhead at water depth of 1,500 m. Several research groups were involved in investigating the fate of the MC-252 oil using newly advanced molecular tools to elucidate microbial interactions with oil, gases, and dispersant. Microbial community analysis by different research groups revealed that hydrocarbon degrading bacteria belonging to *Oceanospirillales*, *Colwellia*, *Cycloclasticus*, *Rhodobacterales*, *Pseudoalteromonas*, and methylotrophs were found enriched in the contaminated water column. Presented here is a comprehensive overview of the ecogenomics of microbial degradation of MC-252 oil and gases in the water column and shorelines. We also present some insight into the fate of the dispersant Corexit 9500 that was added to aid in oil dispersion process. Our results show the dispersant was not toxic to the indigenous microbes at concentrations added, and different bacterial species isolated in the aftermath of the spill were able to degrade the various components of Corexit 9500 that included hydrocarbons, glycols, and dioctyl sulfosuccinate.

## Deepwater Horizon Oil Spill

In April 2010, high-pressure oil and gas caused the Deepwater Horizon drilling rig in the Gulf of Mexico to explode making it the worst oil spill in the United States and the largest marine oil spill in the history of the petroleum industry. MC-252 oil was released from the broken riser pipe at a depth of 1,500 m below surface. Approximately 4.1 million barrels of light crude oil was released into the Gulf waters, of which a significant amount has been accounted for in the cleanup effort (including siphoning, controlled burns, skimming, booming; Atlas and Hazen, 2011). The remaining fraction along with the added dispersants, contributed to the sudden influx of aliphatic and aromatic hydrocarbons in the Gulf water leaving a plume (cloud of dispersed oil droplets), more than 35 km in length (Camilli et al., 2010), that significantly impacted indigenous microbial population. Some fraction of the oil eventually made its way to the beaches, marshes, and sediments. Results from several research groups indicated that the oil degrading indigenous microbes played a significant role in reducing the overall environmental impact of the oil spill (Hazen et al., 2010; Valentine et al., 2010; Atlas and Hazen, 2011; Redmond and Valentine, 2011; Mason et al., 2012).

### Microbial response to MC-252 oil in the deep ocean waters

As the mixture of aromatic (monocyclic and polycyclic) and aliphatic hydrocarbons in MC-252 oil moved through the water column, it was subjected to chemical and physical partitioning. A recent study combining atmospheric, surface, and subsurface chemical data has stated that the readily soluble hydrocarbon components constituted approximately 70% of the deep plume mass and that the remaining traveled as trapped oil droplets throughout the water column (Ryerson et al., 2012). As the spill events progressed, the microbial community changed in response to the available hydrocarbons. Shortly after the spill in May 2010, bacterial counts in the plume were significantly higher (5.5 × 10^4^ cells/mL) than outside the plume (approximately 2.7 × 10^4^ cells/mL; Hazen et al., 2010). The rapid response of different groups of bacteria may imply differential utilization of nutrients/hydrocarbons introduced by the spill (Atlas and Hazen, 2011; Valentine et al., 2012). In the initial stages in May and June 2010, microbial community composition in the plume waters was highly enriched in *Gammaproteobacteria* (Hazen et al., 2010; Redmond and Valentine, 2011). 16S ribosomal RNA based PhyloChip microarray and 16S rRNA gene based clone libraries identified *Oceanospirillales* as dominant microbes (Hazen et al., 2010) but also found 15 other *Gammaproteobacteria* taxa that were enriched by the subsurface plume. Functional gene based GeoChip microarray analysis revealed significant increases in expression of more than 1600 genes involved in hydrocarbon degradation (BTEX, alkane, cycloalkanes, and PAH) over background non-plume samples. In addition, genes for carbon metabolism, nitrogen assimilation, sulfate reduction, phosphorus release, metal resistance, and bacteriophage replication were higher in abundance in plume waters, along with several functional genes derived from *Oceanospirillales*. In absence of an isolate, deep sequencing of community DNA and RNA and single-cell genomics provided greater insights into *Oceanospirillales* for genes and pathway supregulated by the spill (Mason et al., 2012). Genes involved in alkane degradation (specifically, cyclohexane) were expressed along with genes for nutrient uptake, motility, and chemotaxis. Together, they might have enabled cells of this microbial group to colonize, feed on the oil, and multiply in numbers in the plume (Mason et al., 2012).

Clone library analysis of 16S rRNA gene showed dominance of several sequences mostly related to *Cycloclasticus* and *Colwellia* in samples collected from the deep plume in June 2010 (Valentine et al., 2010; Redmond and Valentine, 2011). Stable isotope probing experiments with 13C-labeled gaseous substrates showed that *Colwellia* were likely consuming propane, ethane, and potentially butane. *Cycloclasticus* were thought to be consuming BTEX compounds that were the primary oil constituents found in the subsurface plume during this time period (Redmond and Valentine, 2011). 16S rRNA gene cloning and sequencing of plume samples collected in September 2010 by Kessler et al. (2011) reported a very different microbial community structure, containing high numbers of methylotrophic bacteria (*Methylococcaceae*, *Methylophaga*, and *Methylophilaceae*) and low abundance of *Colwellia*, *Cycloclasticus*, and *Oceanospirillales* – which had dominated previously. The authors attributed this change to a residual bloom of methanotrophic activity having occurred in July 2010. 16S rRNA clone libraries identified methanotrophs, methylotrophs, *Flavobacteria*, and *Alphaproteobacteria* (*Rhodobacterales*) to be relatively abundant in plume waters sampled later in September 2010 (Redmond and Valentine, 2011).

In contrast to bacterial community composition, archaeal community in plume samples did not show much deviation from May through September 2010 (Redmond and Valentine, 2011). Marine Group II *Euryarchaeota*, other marine *Euryarchaeota*, and *Thaumarchaeota* were consistently present, and it is unlikely these archaeal groups had any role in degradation of the oil hydrocarbons (Redmond and Valentine, 2011). Moreover, ammonia oxidation and nitrification by *Nitrosopumilus* *maritimus* belonging to marine Group I *Thaumarchaeota* was only slightly impaired when amended with 10 or 100 ppb oil (Urakawa et al., 2012). These results suggest that the sudden outpouring of oil hydrocarbons from the spill had no significant effect on the marine archaeal population.

Seventeen *Vibrio* isolates representing five distinct genotypes were isolated in April and May 2010 from ocean water, sediment, and oysters in coastal Louisiana (Smith et al., 2012). While some of these *Vibrio* isolates grew on surfactants Tween 40 and Tween 80, none of them were able to use PAH such as naphthalene and phenanthrene (Smith et al., 2012). *Vibrio* strain S4BW isolated from the surface waters 6 weeks after the spill (Gauglitz et al., 2012) was able to produce siderophores to better sequester limiting nutrients like Iron. Several *Vibrio* species belonging to *Gammaproteobacteria* were also isolated from plume water and contaminated Elmers beach samples (Chakraborty, R., unpublished). DNA based dot blot hybridization using a specific probe detected greater than 10^5^ CFU/g of *Vibrio* *vulnificus* in tar balls and sands from beaches in Mississippi and Alabama collected from July through October 2010 (Tao et al., 2011). *Vibrios* are common inhabitants of ocean water (Grimes et al., 2009) and have been associated with hydrocarbon degradation (Hedlund and Staley, 2001; Thompson et al., 2004). Although they are ubiquitous in marine environments, it is unlikely that they were major players in the biodegradation of the oil as they were not amongst the most enriched microbes in the plume or in the coastal contaminated area.

### Microbial response to MC-252 oil in laboratory incubations

By stable isotope probing experiments with 13C-labeled methane, ethane, propane, or benzene in laboratory incubations followed by 16S rRNA gene clone libraries, Redmond and Valentine (2011) demonstrated that *Colwellia* increased in abundance during enrichment on these gases at cold temperatures (4°C). This implied that temperature was a major determinant in selection of this group of microorganisms in the plume. Further, laboratory-based incubations with MC-252 oil, Corexit, and water outside the plume also revealed an increase in abundance of *Colwelliaceae* and *Oceanospirillales* (Bælum et al., 2012). *Colwellia* strain RC25 was isolated from these laboratory incubations that rapidly degraded 75% of the initial oil added in 10 days. 16S rRNA gene sequence of this strain showed 96% sequence similarity to the type strain, *Colwellia* *psychrerythraea* 34H and almost 99% similarity to the most abundant *Colwellia* species observed by 16S pyrosequencing in the original incubations. Interestingly, in these incubations, large flocs seemed to form with oil and/or Corexit, and detailed investigation indicated that *Colwelliaceae* were the dominant bacteria in the flocs. Flocs were absent from incubations amended with Iron. Apart from biomass, flocs contained oil and carbohydrates as revealed by Synchrotron radiation-based Fourier transform infrared (SR-FTIR) spectromicroscopy (Bælum et al., 2012). Flocs were also similarly observed from subsurface plume samples (Hazen et al., 2010) and on deepwater coral colonies near the Macondo well in November and December 2010 (White et al., 2012). It is likely that flocs contained oil, biomass, products of oil degradation, and other carbohydrates such as exopolysaccharides (Bælum et al., 2012; White et al., 2012).

### Microbial response to MC-252 oil in surface waters

Surface water collected in June 2010 about 2–7 km away from the wellhead showed high microbial respiration, high hydrocarbon degradation and high rates of lipase, and alkaline phosphatase activity (Bethanie et al., 2011). Alkaline phosphatases are usually produced by microbes when challenged with phosphate starvation. Nitrate and Phosphate were added to water incubations to alleviate nutrient stress, and this seemed to increase microbial respiration and biomass (Bethanie et al., 2011). Clone library analysis of surface water collected in May and June 2010, 2–44 km from the Macondo wellhead demonstrated that microbial community composition differed dramatically from the deepwater plume sampled in the same time frame (Redmond and Valentine, 2011). *Cyanobacteria* and *Alphaproteobacteria* (SAR11 clade, *Rhodobacterales*, and *Rhodospirillales*) inhabited the surface waters with visible oil sheen, and *Gammaproteobacteria* (*Pseudoalteromonas*, *Pseudomonas*, *Vibrio*, *Acinetobacter*, and *Alteromonas* genera) were prevalent in samples that had heavy oil layer on top. Several *Cyanobacteria*, *Rhodobacterales*, and *Rhodospirillales* have been associated with oil hydrocarbon degradation (Brakstad and Lødeng, 2005; Hernandez-Raquet et al., 2006; Ibraheem, 2010), and several members of these bacterial groups are also capable of photosynthesis. Thus it is no surprise that they were abundant in the surface water. *Oceanospirillales*, *Colwellia*, and *Cycloclasticus*, which were the most enriched microbial group in the deepwater plume samples (Hazen et al., 2010; Valentine et al., 2010; Redmond and Valentine, 2011; Mason et al., 2012) were an insignificant part of the total microbial community in these surface waters.

### Microbial response to MC-252 oil in shorelines and marshes

A considerable amount of oil resulting from the spill impacted coastal waters of the Gulf of Mexico and washed ashore the marshes and the beaches (Allan et al., 2012). To better understand biodegradation of weathered and fresh oil in these environments, indigenous prokaryotic and eukaryotic microbial communities were studied. 18S rRNA gene based phylogenetic analysis was used to characterize eukaryotes inhabiting beach sediments prior to and following shoreline oiling (Bik et al., 2012). In this study, a substantial shift in communities between pre-spill and post-spill was reported. While sediments on the outer shores of Dauphin Island were greatly dominated by *Cladosporium* species (which can utilize hydrocarbon compounds extensively), marine *Alternaria* species dominated in brackish Mobile Bay waters. In addition, OTUs related to *Aspergillus*, *Acremonium*, *Acarospora*, *Rhodocollybia*, and *Rhizopus* species were reported in higher abundance in the post oil spill samples. A number of these marine fungal groups have been shown previously to metabolize hydrocarbon compounds (Bik et al., 2012) similar to those present in MC-252 oil as well.

In a study by Horel et al. (2012), mesocosms were initiated with MC-252 oil and sand from Dauphin Island to mimic the effect of oil spill on sandy beaches. The results confirmed that indigenous hydrocarbon degrading bacteria were present in beach sand and that the rate of oil degradation by these microbes were stimulated when amended with inorganic nutrients.

Culture-dependent and genomics-based studies were used to characterize microbial community in oil-contaminated and pristine sand samples collected from Pensacola beach in September 2010. Oil tainteds and harbored a higher microbial count of hydrocarbon degraders compared to pristine samples as corroborated by both Most Probable Number (MPN) and molecular methods. Based on initial DNA fingerprinting analysis, detailed Pyrotag sequencing of SSU rRNA amplicons revealed a significant community shift toward *Gammaproteobacteria*, and to a lesser extent the *Alphaproteobacteria*, following exposure to oil (Kostka et al., 2011). Furthermore, members of *Alcanivorax*, *Marinobacter*, *Vibrio*, *Pseudomonas*, *Pseudoalteromonas*, and *Acinetobacter* genera were isolated from the contaminated sample, several of which are known hydrocarbon degraders. In MC-252 oil-contaminated samples from Elmer’s Beach, several such hydrocarbon degrading bacteria were isolated with representatives from the *Alcanivorax*, *Marinobacter*, *Pseudomonas*, *Roseobacter*, *Rheinheimera*, and *Vibrio* genera (Chakraborty R, unpublished). Metagenomic and metatranscriptomic analysis revealed an enrichment of a group of organisms within the *Rhodobacterales* family corresponding to samples with high total petroleum hydrocarbons (TPH; Lamendella, R., unpublished).

Sediment from coastal salt marsh in Alabama collected in June, July, and September 2010 were analyzed for structure and function of the native microbial community using PhyloChip and GeoChip microarrays (Beazley et al., 2012). Higher oil concentrations in samples from June and July corresponded to an increase in *Actinobacteria*, *Bacteroidetes*, and *Proteobacteria*. *Firmicutes* like *Bacilli* and *Clostridia* were more abundant in September when oil concentrations were lower. Oil concentration also influenced the community function, as the relative abundance of hydrocarbon degrading genes increased significantly when TPH concentrations were high, and decreased when hydrocarbons were low (Beazley et al., 2012).

Together, these data provide evidence that there exists an active aerobic microbial community indigenous to the shoreline environments that is capable of degrading petroleum hydrocarbons. In the event of an influx of these substrates, *in* *situ* microbial degradation is stimulated and mineralization of hydrocarbons is observed leading to natural attenuation. Rapid microbial respiration of readily accessible substrates also leads to oxygen-depleted subsurface environments. A study by Boopathy et al. (2012) with marsh sediments from Barataria Bay in Louisiana showed that microbial degradation of MC-252 oil occurred under anaerobic conditions as well, although the microbial community involved in the process was not discussed. The presence of *Rhodocyclaceae*, *Geobacteraceae*, and *Desulfobacteraceae* (as shown by 16S rRNA based PhyloChip) along with the detection of genes involved in anaerobic metabolism such as sulfate reduction, nitrate reduction, and methanogenesis (as shown by functional gene microarray, GeoChip), also hinted toward occurrence of anaerobic hydrocarbon degradation in salt marsh sediments in coastal Alabama (Beazley et al., 2012).

## Microbial Response to the Dispersant Corexit 9500

To prevent oil slicks, 1.8 million gallons of the dispersant Corexit 9500 and later Corexit 9527 was used both on surface and at the leaking wellhead. Most commercial dispersants typically contain one or more surfactant(s), with both hydrophilic and hydrophobic groups that encourage development of small oil-surfactant micelles (Hemmer et al., 2011). The resulting greater surface area enhances their entrainment in the water column and enhances bioavailability for microbial degradation while distributing the oil to lower concentrations aided by recirculation of water. However, while the concentrations and dispersant-to-oil ratios used in the MC-252 oil spill were reported to be much lower than the concentrations used for toxicity screenings (Kujawinski et al., 2011), not much is known about the persistence, toxicological effects, and the cumulative impact of dispersant with oil in Gulf of Mexico. In previous studies with microbial consortia at 8°C, addition of Corexit 9500 to fresh or weathered oil had not shown any change in oil degradation (Lindstrom and Braddock, 2002). However, microbial heterotrophs were present in significantly higher numbers in the presence of the dispersant, suggesting that Corexit 9500 provided an additional carbon source. Furthermore, phenanthrene was mineralized better when Corexit 9500 was added (Lindstrom and Braddock, 2002).

In the aftermath of the Deepwater Horizon oil spill, the effect of Corexit 9500 on bacterial viability was studied using isolates obtained from oil-contaminated sands from Elmers Island by Hamdan and Fulmer (2011). In general, Corexit 9500 decreased cell viability at all concentrations tested. At extremely low concentration (dilution of 1:1000), the dispersant seemed to promote cell viability of an isolate 99% similar to *Vibrio* *natriegens* strain UST040801-014. At Corexit dilutions of 1:10, 1:25, and 1:50 (diluted with hexadecane), two hydrocarbon degrading isolates *Acinetobacter* *venetianus* and *Marinobacter* *hydrocarbon oclasticus* were severely affected, however the isolate most similar to *Pseudomonas* *pseudoalcaligenes* showed almost as much growth as the control at 1:50 dilution. Perhaps the presence of hexadecane helped alleviate the toxic effect of the dispersant in this case (Hamdan and Fulmer, 2011).

Corexit 9500 was composed of a mixture of hydrocarbons (50%), glycols (40%), and dioctylsulfosuccinate (DOSS; 10%). Not only did Corexit 9500 have no effect on the growth of microbial consortia enriched from the Gulf of Mexico, most of its components were biodegraded over time – the hydrocarbon fraction much more rapidly than the DOSS and glycol compounds (Bælum et al., 2012). *Colwelliaceae*, *Rhodobacteraceae*, *Oceanospirillales*, and *Actinobacteria* dominated the microbial community in these incubations containing 100 ppm MC-252 oil and 60 ppm Corexit 9500 from which *Colwellia* strain RC25 was isolated. Apart from this study with laboratory microcosm from our group (Bælum et al., 2012), there have been no other reports on the effect of Corexit 9500 on microbes such as *Colwellia*, that played a critical role in responding to the Deepwater Horizon oil spill.

**Figure 1:**
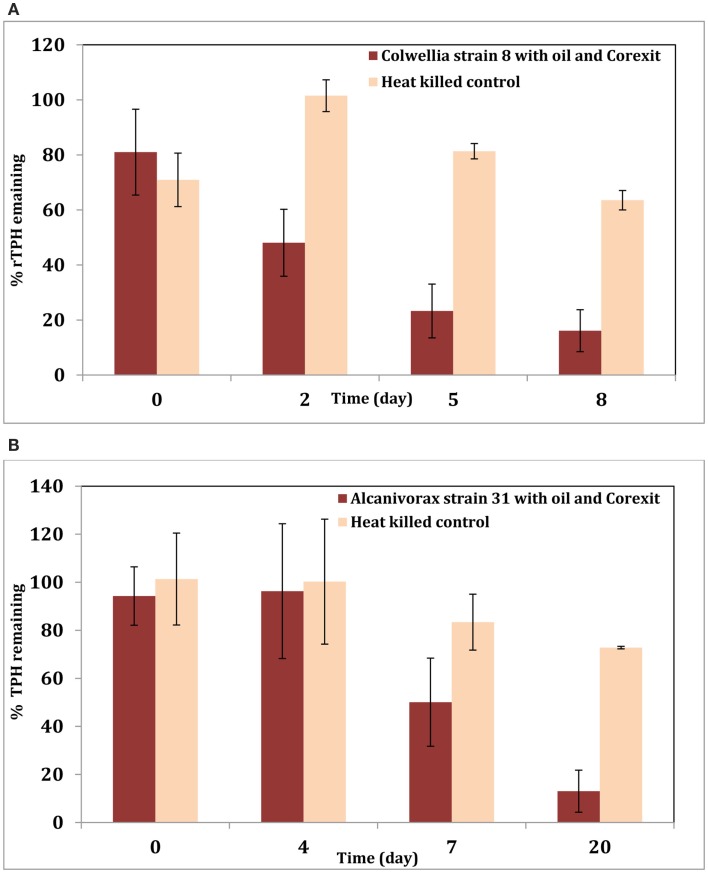
**(A)** Degradation of total petroleum hydrocarbon (TPH) by *Colwellia* strain RC25 with 100 ppm MC-252 oil and 10 ppm Corexit 9500. **(B)** Degradation of TPH by *Alcanivorax* strain 31 with 20 ppm MC-252 oil and 1 ppm Corexit 9500.

To better understand the effect of the dispersant on this organism, experiments were initiated with active cultures of *Colwellia* strain RC25 in minimal media in the presence of MC-252 oil (100 ppm) or MC-252 oil (100 ppm) + Corexit 9500 (10 ppm) at 4°C. Sacrificial samples were analyzed for TPH as described previously (Hazen et al., 2010; Bælum et al., 2012). Results indicate that oil was degraded faster in the treatments with dispersant (Figure 1A). This could be due to the fact that Corexit increases the solubility of oil in water and thus oil is more bioavailable for biodegradation. Similar results were obtained with an *Alcanivorax* strain isolated from oil-contaminated Elmers beach (Figure 1B) when tested with MC-252 oil (20 ppm) with or without Corexit 9500 (1 ppm). These two isolates belong to *Colwelliaceae* and *Oceanospirillales* respectively, which were the dominant microbial groups observed in the oil plume shortly following the spill, and our results confirm their role in biodegradation of the oil promoted by the addition of dispersant. In both experiments, oil (and Corexit) was the sole source of carbon.

**Figure 2:**
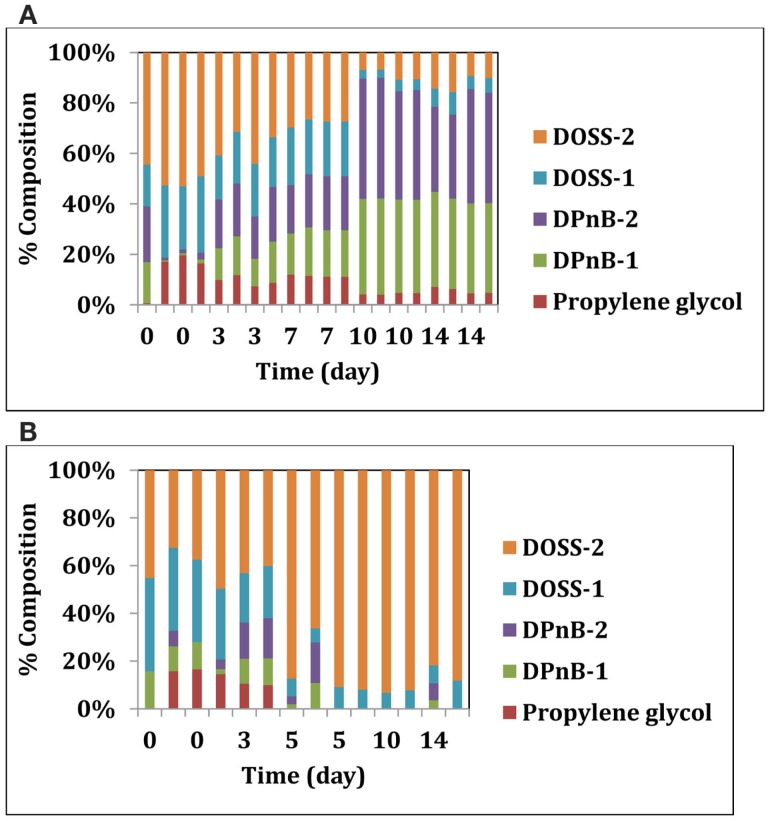
**(A)** Degradation of Corexit 9500 compounds by *Colwellia* strain RC25. **(B)** Degradation of Corexit 9500 compounds by *Alcanivorax* strain 31.

We also looked deeper into the biodegradability of the dispersant itself by these two isolates. Corexit 9500 components were analyzed over time in sacrificial samples using methods described previously (Bælum et al., 2012). Five main compounds were quantified from Corexit 9500: propylene glycol, two isomers of dipropylene glycol n-butyl ether (DPnB), and two isomers of DOSS. Both microbial isolates were able to degrade some components of Corexit 9500 (Figures 2A,B). While the glycol compounds DPnB present in Corexit 9500 were not degraded by *Colwellia* strain RC25, some isomers of DOSS were degraded within 19 days (Figure 2A). In support of this, a slow degradation of DOSS compounds was observed at plume depth after the oil spill (Kujawinski et al., 2011). *Alcanivorax* strain 31 was unable to degrade the DOSS components, but could degrade DPnB and propylene glycol components (Figure 2B). Differential degradation of the various components of Corexit 9500 by these two microbes suggests that complete mineralization could have been possible by consortia of the indigenous microbes that were enriched in the plume.

## Conclusion

A systems biology approach closely integrated with chemical and statistical analyses fueled by interest from the scientific community, regulating agencies, and general public led to an unprecedented near real-time understanding of the fate of MC-252 oil degradation in the Gulf of Mexico (Chakraborty et al., 2012). The rapid response by the scientific community was greatly successful in documenting a comprehensive sequence of events resulting from the Deepwater Horizon oil spill. It was evident that microbes indigenous to the Gulf of Mexico waters were highly capable of mineralizing oil, and groups of microbes capable of degrading certain components of the oil hydrocarbons bloomed in sequence when those hydrocarbons were made available as substrates to them. Natural attenuation was partly facilitated by the addition of dispersant that increased the bioavailability of oil. While the dispersant was detrimental to the survival and health of different macro-organisms, representative microbes enriched from the plume were able to degrade oil better in its presence, and could further degrade certain components of the dispersant as well. Application of traditional microbiological methods with modern genome-based technologies led to an extensive understanding of how the deep-sea and shoreline microbial community responded. This provided an excellent opportunity for the scientific community to be able to predict microbial involvement in major oil spills in future.

## Conflict of Interest Statement

The authors declare that the research was conducted in the absence of any commercial or financial relationships that could be construed as a potential conflict of interest.
